# Food for thought: probiotic modulation of microglial activity in Parkinson's disease

**DOI:** 10.3389/fnmol.2025.1690507

**Published:** 2025-10-09

**Authors:** Isabella Kroker Kimber, Marie-Ève Tremblay

**Affiliations:** ^1^Faculty of Medicine, University of British Columbia, Vancouver, BC, Canada; ^2^School of Medical Sciences, Faculty of Health, University of Victoria, Victoria, BC, Canada

**Keywords:** Parkinson's disease, gut-brain axis, probiotics, neuroinflammation, microglia

## Abstract

The gut-brain axis is emerging as a key player in Parkinson's disease (PD), with growing attention on how the gut microbiome (GM) shapes microglial activity, a central driver of neuroinflammation and dopaminergic loss. GM dysbiosis, characterized by reduced beneficial microbes and increased proinflammatory taxa, can compromise intestinal barrier integrity, activate systemic immunity, and prime microglia toward a proinflammatory state, potentially facilitating α-synuclein misfolding and propagation from gut to brain. Preclinical studies reveal that probiotics can rebalance microbial communities, enhance short-chain fatty acid production, reinforce intestinal barrier integrity, and modulate immune responses, effects collectively linked to reduced microglial reactivity, lower α-synuclein aggregation, and improved motor outcomes in PD models. Human trials of probiotic supplementation in PD, primarily investigating gastrointestinal and non-motor symptoms, suggest potential benefits for systemic inflammation and neuroimmune signaling, though direct evidence of central microglial modulation is limited. By synthesizing animal and clinical data, this review underscores both the therapeutic promise of probiotics and identifies current gaps in leveraging microbiota-based interventions as non-invasive, disease-modifying strategies for PD.

## Graphical Abstract

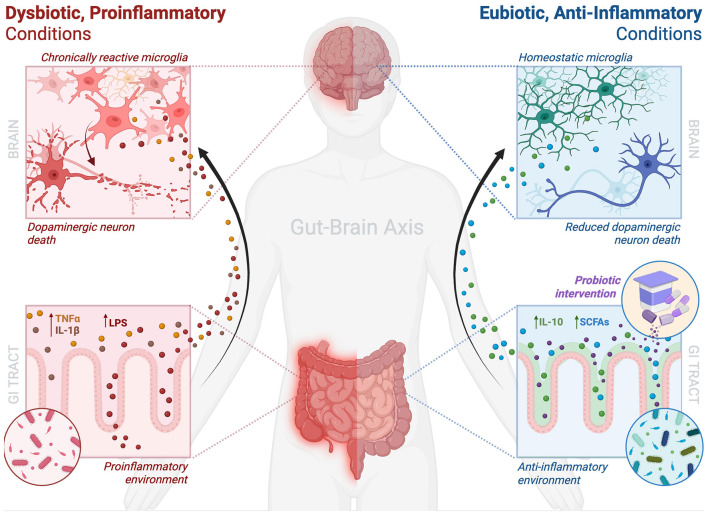

## 1 Introduction

The human microbiome is a dynamic ecosystem composed of tens of trillions of microorganisms, the majority of which reside in the gastrointestinal (GI) tract, collectively forming the gut microbiome (GM) (Bidell et al., 2022; Sender et al., 2016). Initially recognized for metabolic and digestive roles, the GM influences additional physiological processes such as immune system development and brain function, impacting cognition and neural activity through the gut-brain axis (GBA) (Sanz et al., 2025; Zhang et al., 2025).

Of the GBA's various communication pathways, one connection of increasing interest is the influence of the GM on microglia, the brain's resident immune cells. Microglia are critical regulators of brain health. They sculpt neural circuitry and prune synapses especially during development, and throughout life they continuously survey the central nervous system (CNS) for signs of injury or infection (Augusto-Oliveira et al., 2019; Prinz et al., 2019; Thion et al., 2018a). Importantly, microglia regulate neuroinflammation—a CNS immune response involving glial cells, the release of inflammatory mediators, and the synthesis of reactive oxygen species (ROS) and nitric oxide (NO) in response to trauma, infection, or neurodegenerative disease (Adamu et al., 2024). Research has revealed that microglial development, maturation, and reactivity (marked by upregulation of inflammatory markers and morphological changes such as thicker processes and amoeboid-shaped soma) are governed not solely by local CNS signals but also by cues originating in the gut such as short-chain fatty acids (SCFAs) which are vital microbial metabolites produced by gut bacteria through the fermentation of dietary fibers (Abdel-Haq et al., 2019; Cao et al., 2025; Jurga et al., 2020; Mallick et al., 2024; Silva et al., 2020).

Given their central role in neural health, microglia are increasingly implicated in neurodegenerative diseases such as Parkinson's disease (PD) (Bloem et al., 2021; Gao et al., 2023). PD, a progressive disorder projected to more than double by 2050, is influenced by increasing age and sex, with men consistently being at higher risk (Ben-Shlomo et al., 2024; Ou et al., 2021). Characterized by both motor symptoms (bradykinesia, resting tremor, rigidity, postural instability) and prodromal non-motor symptoms (constipation, cognitive impairment, depression), PD results from the pathogenic loss of dopaminergic neurons in the midbrain (substantia nigra pars compacta) and the toxic accumulation of misfolded alpha-synuclein (αSyn) in Lewy bodies (Bloem et al., 2021; Brás and Outeiro, 2021). Neuroinflammation, particularly from chronically dysregulated microglia, plays a central role in PD progression and genome-wide association studies further implicate microglia by identifying PD risk variants enriched in microglial genes, such as LRRK2, HLA-DRB5, and CD33 (Gelders et al., 2018; Guillot-Sestier and Town, 2018; Masuda et al., 2020; Tansey and Romero-Ramos, 2019).

Probiotics, live microorganisms that can confer health benefits, have gained interest as GBA modulators (Bi et al., 2023; Rosas-Sánchez et al., 2025). By alleviating microbiota dysbiosis (reduced microbial diversity and/or overgrowth of harmful bacteria) and increasing beneficial microbial metabolites, such as SCFAs like butyrate, probiotics may influence microglia by modulating peripheral immune signals, blood–brain barrier (BBB) integrity, and inflammatory microglial gene expression (Alagiakrishnan et al., 2024; Bi et al., 2023). In animal models, specific probiotic strains may even reduce microglial reactivity and proinflammatory cytokines in the brain, highlighting their potential to mitigate neuroinflammatory pathways in neurological diseases (Parra et al., 2023; Sun et al., 2021; Tsao et al., 2024).

PD represents a compelling case to explore the probiotic-microglia influence given its rising global incidence, prevalent prodromal gut-related symptoms, and largely idiopathic nature. Thus, this mini review synthesizes current evidence linking probiotic interventions to microglial modulation in PD, highlighting emerging therapeutic possibilities within the GBA. Given limited human studies in this area, findings from mammalian models are also considered to provide a comprehensive overview and guide future preclinical and clinical research directions.

## 2 Gut-brain mechanisms of communication

The human gut harbors an incredibly diverse community of bacteria, fungi, parasites, viruses and protozoa that form the GM and contribute to the synthesis of bioactive compounds such as SCFAs, neurotransmitters, tryptophan metabolites, and select vitamins, all essential to host physiology (Pedroza Matute and Iyavoo, 2023; Thursby and Juge, 2017). Microbiota homeostasis is critical for both GM and host health (Khalil et al., 2024). While considerable inter-individual variation exists, research has attempted to identify common microbial patterns associated with health and disease (Pedroza Matute and Iyavoo, 2023). For instance, physiological stress has been correlated with decreased *Lactobacilli* spp., and reduced Faecalibacterium prausnitzii has been found in patients with Crohn's disease (Bhattarai et al., 2017; Sokol et al., 2008). Unfortunately, no universally consistent microbial signature has been established as a reliable biomarker for disease. This is likely due to the GM's dynamic, complex nature which is shaped by many factors such as diet, environment, genetics, and lifestyle, ultimately complicating causal inferences (Duvallet et al., 2017; Lloyd-Price et al., 2016).

The GBA is a widely studied model for understanding how microbiota shifts influence CNS health. Communication along this axis occurs in a multifaceted manner with three key mechanisms mediating the GM's impact on the brain: immune modulation by microbial products such as SCFAs and lipopolysaccharides (LPS), neural signaling via the vagus nerve, and direct action of microbial metabolites (Fung et al., 2017; Sharon et al., 2016). SCFAs, butyrate being the most well studied, modulate microglial activity and promote anti-inflammatory states, while microbial tryptophan metabolites influence serotonergic signaling and CNS immune tone (Abdel-Haq et al., 2019; O'Mahony et al., 2015). LPS, an outer membrane component of Gram-negative bacteria, can enter systemic circulation during dysbiosis, activating peripheral immunity and promoting neuroinflammation and microglial priming (Banks, 2015; Zhao et al., 2017). Finally, the vagus nerve provides a direct gut-brain conduit, with subdiaphragmatic vagotomy eliminating behavioral and neurochemical effects of certain probiotics in mice, underscoring its role in transmitting microbial signals (Bravo et al., 2011; Carabotti et al., 2015; Zhang et al., 2020). Notably, this communication is bidirectional: the GM influences brain function, while neurological and psychological states, via stress hormones and autonomic signaling, shape the gut environment (Shekarabi et al., 2024).

Among the many CNS components influenced by gut-derived signals, microglia act as a key interface between the gut and the brain. Beyond synaptic remodeling, immune surveillance, and neuroinflammatory responses, evidence from emerging mammalian and human studies suggest that GM-derived signals shape microglial maturation and function, in health and disease (Abdel-Haq et al., 2019; Thion et al., 2018b). In germ-free (GF) mice—raised without a gut microbiota—microglia develop abnormally, with altered morphology, gene expression, and impaired immune responses. Interestingly, these deficits can be partially reversed by colonization with a conventional microbiota or supplementation with SCFAs, suggesting that gut-derived signals are not merely permissive but actively instructive for proper microglial function (Abdel-Haq et al., 2019; Erny et al., 2015).

## 3 The role of microglia across development and adulthood

Microglia are highly specialized immune cells that enter the CNS early in embryonic development and persist as resident cells throughout life (Prinz et al., 2019). Originating from yolk sac progenitors, they populate the brain prior to the formation of the BBB and continuously survey the CNS microenvironment (Prinz et al., 2019; Tay et al., 2017). In addition to maintaining homeostasis, supporting neuronal development and synaptic pruning, microglia are dynamic integrators of peripheral signals, including those from the GM (Wang et al., 2018). Their capacity to respond adaptively to both endogenous and exogenous stimuli allows microglia to maintain CNS health under physiological conditions and mount appropriate responses to injury or disease (D'Alessandro et al., 2022; Salter and Stevens, 2017).

Neuroinflammation is a common feature across many neurodegenerative disorders, including PD. In this context, microglia are considered as key orchestrators. When exposed to stimuli such as misfolded proteins like αSyn, environmental toxicants, systemic inflammation, or foreign microbial metabolites, microglia can shift into a proinflammatory phenotype (Gao et al., 2023; Perry and Teeling, 2013). This is characterized by increased expression of surface receptors (e.g., Toll-like receptor 4 (TLR4)), production of proinflammatory cytokines (IL-1β, TNF-α, IL-6), and generation of ROS and NO, contributing to oxidative stress and neuronal injury (Franklin et al., 2021; Gao et al., 2023). Although acute inflammation aids in repair and pathogen clearance, chronic or inappropriate microglial reactivity can exacerbate neuronal damage (Gao et al., 2023; Ransohoff, 2016).

Importantly, microglia do not operate in isolation. Their inflammatory responses and physiological activities can be shaped by systemic factors, including signals from the GM. As shown in GF mice, microglia exhibit altered morphology and impaired immune responses, effects that can be partially rescued by microbial colonization or SCFA supplementation, supporting the critical role of the GM in neuroimmune homeostasis (Caetano-Silva et al., 2023; Dalile et al., 2019; Erny et al., 2015). Human and translational studies further link gut microbial alterations with changes in neuroinflammatory tone and microglial reactivity across neurological disorders (Morais et al., 2021).

The responsiveness of microglia to microbial signals positions them as a cellular interface of the GBA, allowing gut-derived factors to influence CNS inflammation (Diaz Heijtz, 2016; Sampson and Mazmanian, 2015). In diseases like PD, where chronic microglial reactivity and neuroinflammation are prominent, understanding upstream modulators of microglial function, including the GM, may offer new therapeutic avenues (Wang et al., 2018). As such, microglia represent not only a pathological hallmark but also a potentially modifiable target within the GBA, especially through interventions like probiotics that aim to correct gut dysbiosis and reduce neuroinflammatory signaling (Zhu et al., 2024).

## 4 Consequences of gut dysbiosis in Parkinson's disease

### 4.1 Gut dysbiosis, barrier dysfunction, and microglial reactivity in Parkinson's disease

GI dysfunction, particularly constipation, is common in the prodromal phase of PD, suggesting a role for the gut in disease onset. In PD, proinflammatory microbial imbalances are thought to compromise gut barrier integrity, alter metabolite production, and disrupt immune signaling, with downstream consequences for CNS function (Cryan et al., 2019; Nie and Ge, 2023). Evidence from animal models supports this link: transplants from PD patients into GF, αSyn-overexpressing (ASO) mice worsen motor deficits and drive microglia toward a proinflammatory state (Cryan et al., 2019; Sampson et al., 2016). In humans, sequencing and metagenomic studies in patients with PD revealed decreased abundance of SCFA-producing genera and increases in proinflammatory taxa (Boktor et al., 2023; Pavan et al., 2023). Across geographically diverse cohorts, these shifts point toward a reproducible dysbiotic signature that may actively contribute to PD pathogenesis, potentially through microglial priming and promoting neuroinflammation.

A key consequence of gut dysbiosis is impaired intestinal barrier integrity. Loss of SCFA-producing microbes, particularly butyrate-producers, can weaken the mucus layer via decreased mucin production—glycoproteins that form the mucus layer to protect intestinal epithelium—and impair tight junction assembly via downregulation of key proteins such as zonula occludens-1 (ZO-1), occludin, and claudin-1 (Di Vincenzo et al., 2024; Kang et al., 2022; Peng et al., 2009; Pérez-Reytor et al., 2021; Wang et al., 2012). This allows for the translocation of microbial metabolites and pathogen-associated molecular patterns (PAMPs) such as LPS into the lamina propria and systemic circulation. There, these molecules engage pattern recognition receptors (PRRs) like TLR4, driving release of proinflammatory cytokines (TNF-α, IL-1β, IL-6) (Di Vincenzo et al., 2024). Resulting peripheral immune activation can prime microglia, lowering their ‘activation' threshold and increasing susceptibility to neuroinflammatory cascades in PD (Chen et al., 2021; Dogra et al., 2021). Supporting this, patients with PD exhibited increased intestinal permeability, reduced colonic ZO-1 expression, elevated mucosal TLR4+ cells, and decreased butyrate-producing bacteria (Perez-Pardo et al., 2019). Further, in a rotenone-induced PD mouse model, TLR4-knockout mice were protected against phagocytic, proinflammatory microglial activity, dopaminergic neuronal loss, and motor impairments, reinforcing the link between gut barrier dysfunction, microglial activity, and neuroinflammation in PD (Perez-Pardo et al., 2019).

Peripheral inflammation affects the brain both directly and indirectly. Circulating cytokines and LPS can cross and potentially disrupt the BBB, altering endothelial and astrocytic modulation and increasing cerebral prostaglandin E_2_ (Gryka-Marton et al., 2025; Varatharaj and Galea, 2017). Similarly, peripheral cytokines enhance BBB permeability and promote endothelial activation, establishing a neuroinflammatory milieu (Varatharaj and Galea, 2017). Microglia are exquisitely sensitive to such inflammatory cues, particularly LPS and TNF-α, which can act on microglia via TLR4, leading to the production of IL-1β, IL-6, TNF-α, and ROS (Walker et al., 2014; Woodburn et al., 2021). This chronically reactive state enhances phagocytosis, antigen presentation, and upregulates CD86 (T-cell co-stimulation) and CD68 (phagolysosomal activity), ultimately promoting dopaminergic neurotoxicity and impaired homeostatic surveillance (Fornari Laurindo et al., 2023). Preclinical studies have demonstrated that peripheral LPS induces microglial reactivity and accelerates dopaminergic cell loss across various PD models while TLR4 inhibition reduces microglial proinflammatory activity and neurodegeneration (García-Domínguez et al., 2018; Perez-Pardo et al., 2019; Xie et al., 2023; Zhang et al., 2022). Further, GF mice, which display immature microglia, regain normal inflammatory responsiveness after colonization with a conventional microbiota or SCFA supplementation, reinforcing the importance of microbial signals in microglial homeostasis (Erny et al., 2015).

### 4.2 α-Synuclein pathology and gut-brain propagation

Alongside neuroinflammation, PD is defined by the aggregation of αSyn into Lewy bodies and neurites (Tansey and Romero-Ramos, 2019; Xiang, 2025). Growing evidence suggests that αSyn misfolding may begin in the gut, having been detected in GI tract and brain of patients with PD prior to onset of motor symptoms (Brás and Outeiro, 2021; Chen and Lin, 2022; de Lataillade et al., 2020; Nie and Ge, 2023). It is hypothesized the GM dysbiosis may drive this process: the loss of anti-inflammatory and SCFA-producing bacteria stresses enteric neurons, inducing αSyn expression, misfolding, and aggregation (Chen and Lin, 2022; Claudino dos Santos et al., 2023). Supporting this, in a rotenone PD model, bacterial endotoxins activated signaling pathways that promoted αSyn aggregation, an effect reversed by antibiotics or fecal transplants from healthy donors (Fang et al., 2024). Notably, LPS may further accelerate this process by binding directly to αSyn and facilitating amyloid fibril formation (Bhattacharyya et al., 2019).

Preclinical and clinical studies provide further support for gut-to-brain spread. Following duodenal human αSyn injections, αSyn fibrils were detected in regions of the vagus nerve connected to the gut in transgenic rats, a process prevented by vagotomy (Kim et al., 2019; Van Den Berge et al., 2019). In human patients, two independent studies of vagotomised patients suggested a reduced PD risk, though long-term follow-up indicated this protection may not be absolute (Liu et al., 2017; Svensson et al., 2015; Tysnes et al., 2015). Together, this research suggests that GM imbalances not only foster αSyn misfolding locally but set the stage for its propagation to the brain.

### 4.3 Clinical implications and therapeutic potential

Recognition of the GBA in PD opens new avenues for clinical intervention, shifting attention toward earlier diagnosis, disease modification, and even, potential prevention. GI αSyn pathology, barrier dysfunction, and gut dysbiosis offer a window into the prodromal phase of PD, sparking interest in identifying early biomarkers of GBA dysfunction, such as altered SCFA levels, increased fecal zonulin (intestinal permeability marker), or loss of butyrate-producing bacteria, that could stratify PD risk or monitor therapeutic response. However, biomarker standardization is lacking and their predictive power for PD progression remains uncertain (Aho et al., 2021; Nie et al., 2022).

Therapeutically, direct manipulation of the GM is under investigation. Beyond current dopaminergic symptom management, mounting evidence suggests that gut-level interventions may reduce systemic inflammation, microglial phenotypic transformation, and αSyn pathology (Alam et al., 2024; Bloem et al., 2021). Probiotics thus offer an attractive approach due to their non-invasive, relatively low-risk, modifiable nature, and potential to reshape microbial ecology and host immune responses. Preclinical and small human studies suggest they may:

- **Restore gut barrier function** by upregulating expression of tight junction proteins like occludin and ZO-1;
- **Reduce systemic and neuroinflammation** by modulating cytokine production and microglial reactivity;
- **Suppress** **αSyn misfolding and propagation** via SCFA-mediated effects on protein homeostasis and enteric neuronal stress (Ahn et al., 2022; Lorente-picón and Laguna, 2021; Zhu et al., 2022).

However, large-scale human trials are needed to determine whether probiotic use can meaningfully alter the trajectory of PD beyond merely GI symptom relief (Leta et al., 2021).

## 5 Probiotics as modulators of microglia in Parkinson's disease

A healthy GM is essential for numerous biological processes, including proper microglial development and function (Erny et al., 2015). In PD, where microglial dysfunction contributes to dopaminergic neurodegeneration, targeting microglia via the GM has gained increasing interest (Alam et al., 2024; Salim et al., 2023). Probiotics offer a promising, non-invasive approach to restoring microbial balance, enhancing gut barrier integrity, and modulating immune and microglial responses, mechanisms thought to affect both the prodromal and symptomatic stages of PD (Leta et al., 2021). Although several clinical trials have evaluated probiotics in PD, most have not directly assessed microglial activity, limiting insight into their central immunomodulatory effects (Magistrelli et al., 2024; Yang et al., 2023; Zali et al., 2024). Consequently, much of our understanding arises from preclinical research. This section highlights key evidence from human and animal studies on probiotics in PD, focusing on how restoring a balanced GM may ameliorate disease symptoms by promoting microglial homeostasis and dampening neuroinflammation.

Animal studies provide compelling evidence that probiotics modulate microglial function and attenuate neuroinflammation in PD models. In ASO mice, prebiotics (non-digestible fibers promoting beneficial bacterial growth) and probiotics reduced microglial reactivity, normalized neuroimmune signaling, and improved motor outcomes (Abdel-Haq et al., 2022; Parra et al., 2023). Abdel-Haq et al. demonstrated that a prebiotic-enriched diet restored microglial homeostasis, reduced αSyn aggregation in the substantia nigra, and improved motor function, effects further negated by microglial depletion, confirming their central role in neuroprotection (Abdel-Haq et al., 2022). In an inflammatory PD rat model (intra-striatal LPS injection), Parra et al. reported that Microbiot^®^ (*Lactobacillus rhamnosus* GG and *Bifidobacterium animalis lactis*) reduced proinflammatory microglial responses, although dopaminergic neuron degeneration persisted (Parra et al., 2023). Similarly, Symprove™, a commercial probiotic formulation, decreased proinflammatory microglial markers, circulating proinflammatory cytokines and LPS, and dopaminergic neuron loss in an early-stage neurotoxin-induced rat model (Sancandi et al., 2023). Sun et al. demonstrated that *Clostridium butyricum* supplementation in MPTP (1-methyl-4-phenyl-1, 2, 3, 6-tetrahydropyridine)-induced PD mice decreased motor deficits and reversed proinflammatory microglial phenotypes and neuroinflammation through GM modulation and enhancing glucagon-like peptide-1 (GLP-1) signaling (Sun et al., 2021). Collectively, these studies indicate that probiotics act via the GBA to shape microglial activity and confer neuroprotection in PD.

Translating preclinical findings to humans remains a challenge, but emerging trials suggest clinical relevance with several reporting improvements in motor symptoms, inflammatory markers, and gut barrier integrity. A randomized controlled trial (RCT) of 128 patients with PD found that *Lacticaseibacillus paracasei* strain Shirota significantly improved constipation and non-motor symptoms without causing major shifts in GM composition (Yang et al., 2023). Notably, fecal L-tyrosine decreased while plasma L-tyrosine increased, suggesting enhanced absorption and potential support for neurotransmitter synthesis (Yang et al., 2023). Another RCT of 40 patients reported that a *Bifidobacterium* cocktail improved motor and non-motor symptoms and reduced plasma proinflammatory cytokines (Magistrelli et al., 2024). Further, a cohort study of 46 patients with PD also found that probiotic supplementation with vitamin D lowered levels of proinflammatory cytokines while improving GI and cognitive symptoms, suggesting a systemic, anti-inflammatory effect of probiotics via the GM (Zali et al., 2024).

Together, these clinical and preclinical findings summarized in Table 1 support the hypothesis that probiotics influence central immune responses through peripheral microbial modulation. While further studies, especially in human populations, are needed, current evidence suggests that targeting microglial dysregulation through the GBA may represent a novel therapeutic strategy to slow neuroinflammation and modify disease progression in PD.

**Table 1:** Overview of preclinical and clinical studies examining probiotics supplementation in Parkinson's disease, with a focus on gut–microglia interactions and neuroinflammatory outcomes.

**Model**	**Intervention**	**Key outcomes**	**Microglia outcome**	**GBA relevance**	**Ref**.
**Preclinical studies**					
αSyn-overexpressing (ASO) male mice	High-fiber prebiotic-enriched diet	Improved motor function; ↓αSyn aggregation in SN	↓ Microglial proinflammatory activity; benefits abolished with microglial depletion	Restored microglial homeostasis via GM; increased major SCFAs	Abdel-Haq et al., 2022
LPS-induced PD male Wistar rat model	Microbiot^®^ (*L. rhamnosus* GG + *B. animalis* ssp. *lactis*)	↓ Striatal microgliosis; no prevention of DA neuron loss	↑ Proportion of homeostatic microglia	Suggests anti-inflammatory effects via GM modulation	Parra et al., 2023
Early-stage 6-OHDA PD male Wistar rat model	Symprove™ (multi-strain probiotic)	↓ Motor deficits; ↓ systemic LPS; ↓ dopaminergic neuron loss	↓ Reactive microglial morphology; ↓ proinflammatory plasma cytokines	Enhanced gut barrier and systemic inflammation control; prevented SCFA decrease	Sancandi et al., 2023
MPTP-induced PD male C57BL/6 mouse model	*Clostridium butyricum*	Improved motor function; ↓ dopaminergic neuron loss; ↑ GLP-1 signaling	↓ Microglial proinflammatory responses; ↓ neuroinflammation	Demonstrates GM-GLP-1 pathway in CNS immune regulation	Sun et al., 2021
**Clinical studies**					
128 PD patient RCT	*Lacticaseibacillus paracasei* strain Shirota fermented milk	Improved constipation and non-motor symptoms	Not investigated	Potential support for neurotransmitter synthesis via GM	Yang et al., 2023
40 PD patient RCT	*Bifidobacterium* probiotic cocktail	↓ Motor and non-motor symptoms; ↓ proinflammatory serum cytokines (IFNγ, IL-6)	Not investigated	Potential influence on neuroimmune interactions via GBA	Magistrelli et al., 2024
46 PD patients (Iranian cohort)	Probiotic cocktail + vitamin D	↓ disease severity, non-motor symptoms; ↓ proinflammatory serum cytokines (IFNγ, IL-6, IL-1β); ↑ anti-inflammatory serum cytokine (IL-10)	Not investigated	Modulates gut–immune signaling; reduces systemic inflammation and enhances gut-mediated immune responses via GBA	Zali et al., 2024

## 6 Discussion

Growing evidence supports the existence of a bidirectional GBA in PD, with gut dysbiosis contributing to or potentially even driving microglial dysfunction and neurodegeneration (Pfaffinger et al., 2025; Xiang, 2025). Preclinical studies demonstrate that probiotics have potential to restore GI eubiosis (a balanced, healthy gut microbiota) and dampen microglial-mediated neuroinflammation, improving motor outcomes and reduced α-synuclein aggregation, a hallmark of PD. However, translating these findings to the clinic remains complex.

A lack of standardization in probiotics research regarding strain specificity, dosage, treatment duration, and outcome measures limits reproducibility, cross-study comparisons and strain-specific effects (Rosas-Sánchez et al., 2025; Smolinska et al., 2025). More studies comparing different types of probiotics are needed to determine which strains ameliorate PD symptoms and to clarify their mechanisms of action on the GBA. Additionally, most clinical trials focus on symptomatic improvements and peripheral immune markers, with few directly assessing microglial activity or central inflammation. Techniques such as positron emission tomography (PET) using TSPO-targeting radioligands (translocator protein notably upregulated in reactive microglia) and emerging radioligands, or magnetic resonance imaging (MRI) sensitive to microglial morphology and neuroinflammatory signatures, could assess microglia-specific changes *in vivo* (Garcia-Hernandez et al., 2022; Guilarte, 2019; Lavisse et al., 2021). Currently, this gap hinders the mechanistic validation of probiotics' neuroimmune benefits in humans, even as peripheral changes suggest indirect support for such effects.

While targeting microglia with probiotics is promising, further high-quality, mechanistically informed trials are needed. Future research should identify reliable, non-invasive biomarkers such as SCFA levels, fecal calprotectin (a marker of intestinal inflammation), or gut permeability markers to detect early GBA dysfunction and monitor therapeutic response (Chai et al., 2025). Emerging neuroimaging techniques and cerebrospinal fluid-based assays may allow researchers to assess microglial activity more directly *in vivo*. Integrative approaches combining probiotics with prebiotics, dietary modulation, or agents like GLP-1 agonists, which enhance gut barrier function and reduce neuroinflammation, may yield synergistic benefits for both gut and brain health (Loh et al., 2024; Menozzi et al., 2025). With appropriate clinical tools and biomarkers, probiotics may one day complement conventional PD therapies by targeting both motor and non-motor symptoms at their neuroimmune root.
